# Economic benefits of blast-resistant biofortified wheat in Bangladesh: The case of *BARI Gom 33*

**DOI:** 10.1016/j.cropro.2019.05.013

**Published:** 2019-09

**Authors:** Khondoker A. Mottaleb, Velu Govindan, Pawan K. Singh, Kai Sonder, Xinyao He, Ravi P. Singh, Arun K. Joshi, Naresh C.D. Barma, Gideon Kruseman, Olaf Erenstein

**Affiliations:** a Socioeconomics Program, CIMMYT (International Maize and Wheat Improvement Center), Carretera México-Veracruz Km. 45, El Batán, Texcoco, Mexico, C.P. 56237; b Global Wheat Program, CIMMYT, Mexico; c Wheat Pathology, Global Wheat Program, CIMMYT, Mexico; d Geographical Information System Unit, CIMMYT Mexico; e Bread Wheat Improvement, Global Wheat Program, CIMMYT, Mexico; f CIMMYT- Borlaug Institute for South Asia (BISA), NASC Complex, New Delhi, India; g Bangladesh Wheat and Maize Research Institute (BWMRI), Bangladesh; h Ex ante and Foresight Specialist, Socioeconomics Program, CIMMYT, Mexico; i Socioeconomics Program, CIMMYT, Mexico

**Keywords:** Adoption, Biofortified, Climate analogue, *Ex-ante*, Food security, Net benefit, Nutrition, Vulnerable, Wheat blast, Zinc enriched

## Abstract

The first occurrence of wheat blast in 2016 threatened Bangladesh's already precarious food security situation. The Bangladesh Agricultural Research Institute (BARI), together with the International Maize and Wheat Improvement Center (CIMMYT) developed and released the wheat variety *BARI Gom* 33 that is resistant to wheat blast and other common diseases. The new variety provides a 5–8% yield gain over the available popular varieties, as well as being zinc enriched. This study examines the potential economic benefits of *BARI Gom 33* in Bangladesh. First, applying a climate analogue model, this study identified that more than 55% of the total wheat-growing area in Bangladesh (across 45 districts) is vulnerable to wheat blast. Second, applying an *ex-ante* impact assessment framework, this study shows that with an assumed cumulative adoption starting from 2019–20 and increasing to 30% by 2027–28, the potential economic benefits of the newly developed wheat variety far exceeds its dissemination cost by 2029–30. Even if dissemination of the new wheat variety is limited to only the ten currently blast-affected districts, the yearly average net benefits could amount to USD 0.23–1.6 million. Based on the findings, international funder agencies are urged to support the national system in scaling out the new wheat variety and wheat research in general to ensure overall food security in Bangladesh and South Asia.

## Highlights

• Emergence of wheat blast in Bangladesh severely threatens South Asia's food security.
• CIMMYT and Bangladesh has developed the first blast resistant wheat in the world.
• The blast resistant BARI Gom 33 is zinc enriched and provides 5–8% yield gain.
• This study examines the economic benefits of the dissemination of BARI Gom 33.
• Analysis shows that the potential economic benefit far exceeds its dissemination cost.

## 1 Introduction

Wheat *(Triticum aestivum* L*.*) is prone to the deadly wheat blast disease caused by *Magnaporthe oryzae* pathotype *triticum* (MoT) which has now emerged in Bangladesh (Callaway, 2016; Islam et al., 2016; Malaker et al., 2016; Mottaleb et al., 2018b), its first occurrence outside of Latin America. In the 2015–16 wheat season, wheat blast affected nearly 15,000 ha in eight districts in Bangladesh (or 3.4% of the domestic wheat-producing area of 445,000 ha in the 2015-16 season). In some districts, up to 70% of wheat-producing land was affected and yield losses were up to 51% in affected fields in some areas (Islam et al., 2016). The disease re-emerged in the 2016-17 season, infecting two additional districts, despite relatively unfavorable weather conditions for disease development: less humidity and relatively cooler weather (CIMMYT, 2017). The application of fungicides is only partially effective to combat wheat blast, and in extreme cases, wheat blast can destroy the entire wheat yield (Urashima et al., 2009).

The emergence of wheat blast in Bangladesh poses a serious threat to South Asia's food security, which is already precarious due to rapid population growth and a strained natural resource base. Wheat blast could spread across the region, starting with neighboring West Bengal, India, which has a similar agro-ecology and shares a 2217-km-long border, and then possibly to relatively warmer wheat-producing regions in Pakistan, such as Sindh. In a recent study, applying the climate analogue analysis, Mottaleb et al. (2018b) warned that out of 40.9 million ha of total wheat-producing land in India, Bangladesh, and Pakistan, more than 17% of the area (6.9 million ha) is vulnerable to wheat blast.

Wheat is the major staple food in northern India and Pakistan (Charath et al., 2007), and has been gaining popularity in Bangladesh, where it is the second major staple after rice (Mottaleb et al., 2018c). Currently, India is a net exporter of wheat, Pakistan is almost self-sufficient, and Bangladesh is a net importer of wheat (FAOSTAT, 2019a). South Asia's population is projected to increase to 2.29 billion by 2050, up from 1.74 billion in 2015 (World Bank, 2019). To meet the growing demand of the burgeoning population, South Asia would need to increase wheat production by 2.25% every year (Charath et al., 2007). This would be thwarted by the further spread of wheat blast, thereby potentially having severe negative impacts on the region's food security and poverty, which already currently sees 16.1% of its population living in extreme poverty (World Bank, 2017). Conversely, successful control or eradication of wheat blast in Bangladesh could generate significant positive externalities across South Asia.

To find a long-term sustainable solution to combat wheat blast, the Bangladesh Agricultural Research Institute (BARI), in collaboration with the International Maize and Wheat Improvement Center (CIMMYT), has developed and tested diverse wheat lines. As part of the quest, *BARI Gom 33* was released in the fall of 2017, as a new blast-resistant wheat for Bangladesh. In addition to being blast-resistant, the variety provides a 5–8% yield benefit and is zinc (Zn) enriched (BARI, 2017).

In a normal release and seed multiplication scenario, it can take at least four to five years for a new wheat variety to reach the masses of farmers in Bangladesh. Normally, in the first two years, the Wheat Research Center (WRC) will produce nucleus and breeder seeds. The Bangladesh Agriculture Development Corporation (BADC) will then further multiply them for another two years to produce certified seeds, which will be subsequently disseminated by the Department of Agriculture Extension (DAE). Given the blast threat, there may be merit in facilitating and ensuring the seed multiplication and dissemination process of the new wheat *BARI Gom 33* at a more rapid rate.

The objective of this study was to quantify the potential economic benefits of the new wheat *BARI Gom 33* in Bangladesh. Examining the potential benefits may help to secure political and financial support, and ensure the timely scale out the new wheat. As the base seed of the new wheat was just made available to the national seed board and is still not in the hands of the farmers, an economic surplus analysis, in the form of the *ex-ante* impact-assessment framework has been applied. Using *BARI Gom 33* in Bangladesh as a case, the present study thus illustrates the importance of the development and rapid dissemination of new biotic stress-resistant crop varieties in developing countries.

The study is organized as follows: Section 2 includes the current state of wheat production and consumption, the emergence of wheat blast in Bangladesh, and the development of the new wheat variety *BARI Gom 33*; Section 3 presents our materials and methods, including identification of wheat blast-vulnerable districts and the *ex-ante* economic quantification framework; Section 4 presents the major findings; and Section 5 concludes and provides policy implications.

## 2 Background and context

### 2.1 Wheat in Bangladesh

Bangladesh is a rice economy, with nearly 75% of its total of 7.92 million hectares of net cropland solely allocated to rice production (BBS, 2018a). Wheat is the second major staple cereal, increasingly cultivated during the mild winter season (November to April) and with a surging consumption demand. The yearly per capita wheat consumption in Bangladesh has increased by 102% from 8.62 kg in 1961 to 17.5 kg in 2013 (FAOSTAT, 2019b). The rapid increase in wheat consumption reflects a higher income elasticity of wheat compared to rice – i.e., with increasing income people consume more wheat relative to rice. In the 2008–09 wheat season, the total land allocated to wheat was 394,619 ha, and the total wheat production in the country was 849,046 metric tons (BBS, 2012). In the 2017–18 wheat season, the area reduced to 351,213 ha: equivalent to less than 3% of Bangladesh's cropland (BBS, 2019), and a 12.3% reduction over the 2008-09 season (Table 1). This reduction in land committed to wheat growing is mainly the result of government policy discouraging wheat cultivation in the severely blast-affected districts following the 2015-16 blast outbreak (Mottaleb et al., 2019a). Note that the government of India is also discouraging wheat cultivation in the border districts of West Bengal; State of India to avoid any possible intrusion of blast pathogen from Bangladesh (Mottaleb et al., 2019b). Total wheat production in 2017–18 was 1.09 million tons, 19% higher than 2008–09 (Table 1). With a domestic price of USD 268/ton, the product value of wheat in 2017–18 was nearly USD 295 million. Bangladesh mostly relies on wheat imports to meet the surging demand, the imports making up nearly 75% of consumption. From 2015–16 to 2017–18, the triennium average shows that Bangladesh imported 5.48 million tons of wheat (Table 1) worth USD 1.5 billion with an import price of USD267/metric ton.

**Table 1:** Wheat area (‘000 ha), yield (metric ton/ha), and production (‘000 metric ton) and selected indicators in Bangladesh and selected districts, 2008–18.

Year	National					Districts not yet affected by blast			10 wheat blast affected districts			45 wheat blast vulnerable districts		
	Area	Yield	Production	Price (USD/ton)^h^	Import (million metric ton)^g^	Area	Yield	Production	Area	Yield	Production	Area	Yield	Production
2008-09^a^	395	2.15	849	176^d^	2.88	256	2.13	544	139	2.20	305	266	2.0	588
2009-10^a^	373	2.60	969	212^d^	3.35	242	2.66	644	131	2.48	325	246	2.04	590
2010-11^a^	374	2.60	972	251^d^	3.95	244	2.52	617	129	2.75	355	239	2.19	632
2011-12^a^	358	2.78	995	241^d^	2.04	227	2.71	614	131	2.91	382	234	2.26	643
2012-13^a^	417	3.01	1255	269^e^	2.73	268	2.95	790	149	3.12	465	273	2.45	825
2013-14^a^	430	3.03	1303	274^e^	3.35	274	2.99	820	156	3.11	483	281	2.48	851
2014-15^a^	437	3.09	1348	256^e^	3.93	279	3.04	848	158	3.17	500	281	2.54	868
2015-16^b^	445	3.03	1348	249^e^	4.72	288	3.04	876	156	3.02	472	277	2.55	827
2016-17^b^	415	3.16	1311	243^e^	5.56	305	3.14	958	110	3.21	354	226	2.67	703
2017-18^c^	351	3.13	1099	268^f^	6.15	305	2.26	947	96	3.13	312	195	2.74	613

Notes: Sources reported per year across all indicators, unless otherwise indicated.

a BBS, (2018b).

b BBS, (2018a).

c BBS (2019).

d (BBS, 2014).

e (BBS, 2018a).

f Department of Agricultural Marketing (2018).

g USDA (2018).

h Currency converted into USD applying exchange rate USD1 = BDT82.

**Fig. 1:**
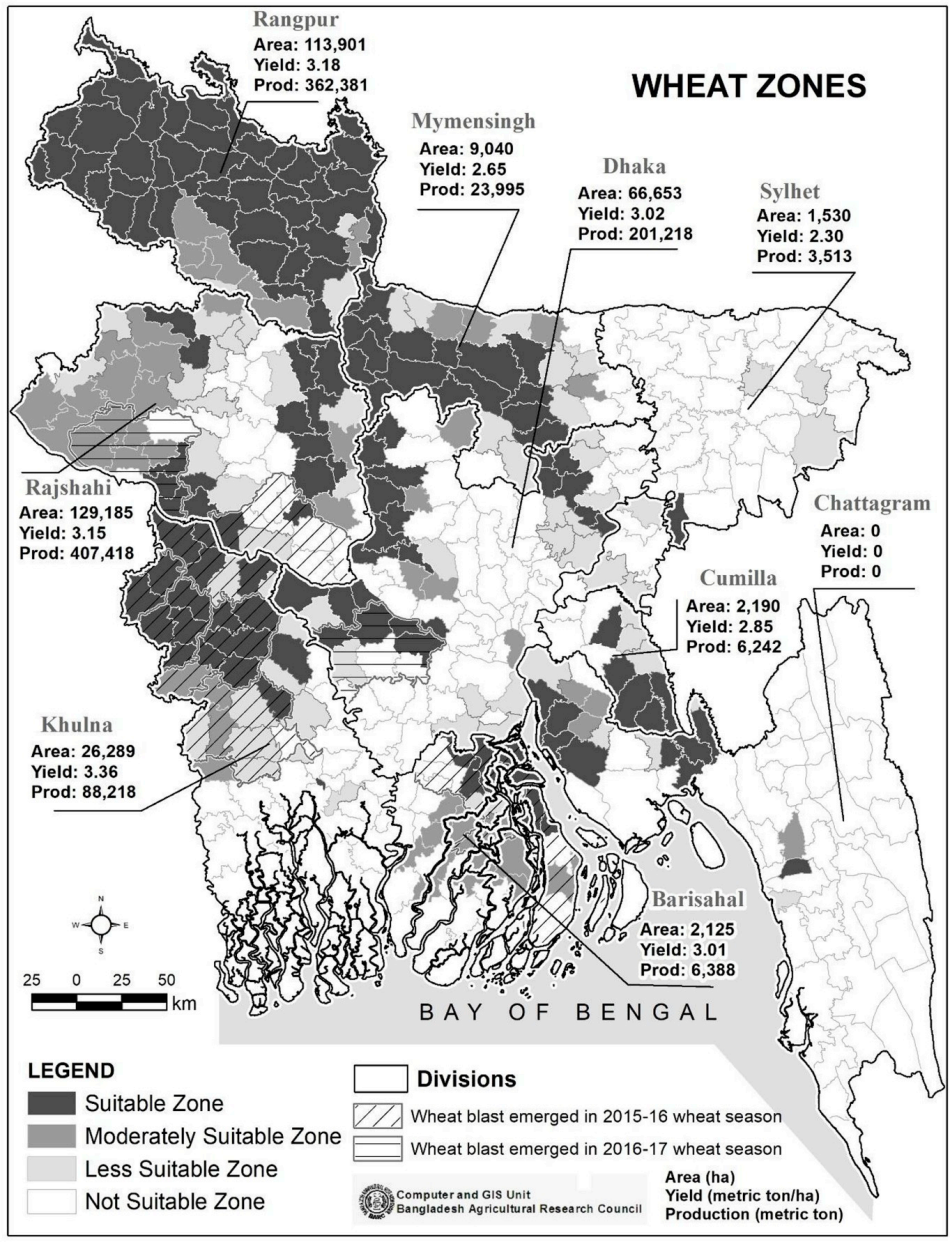
Wheat in Bangladesh: Suitability zones, production statistics by division (2017–18) and wheat blast first incidence (2015–16 or 2016–17). Sources: BARC (2019); BBS (2019); CIMMYT (2017); Islam et al. (2016).

Out of Bangladesh's 64 districts, wheat is currently cultivated in 56 districts (BBS, 2019), but with a concentration in the cooler north and north-west Rangpur and Rajshahi divisions (Fig. 1). In Bangladesh, more than 49% of the total cropland is classified as suitable for wheat cultivation; whereas 25% is moderately-marginally suitable, and 26% of the area is not suitable (BARC, 2019). Wheat blast first emerged in Bangladesh in the 2015–16 wheat season in eight central western districts (Islam et al., 2016, Fig. 1), with two additional districts in the subsequent year. Because of the blast incidence and government discouragement, the 10 wheat blast-affected districts had markedly reduced wheat-growing areas (−12.5%) in the 2017-18 season compared to the previous year (Table 1). The wheat-producing areas in districts not affected by wheat blast still realized a small increase in the same 2016-17 season, but the overall domestic wheat-growing area was reduced by 15.4% compared to the previous year (BBS, 2019, Table 1).

### 2.2 Development of the blast-resistant *BARI Gom 33* wheat variety

CIMMYT works closely with partners from developing countries to develop and disseminate high-yielding, disease-resistant wheat varieties including those with significantly high zinc (Zn) and iron (Fe) concentrations in the grains. Though the major focus is to breed high-Zn-content wheat, the positive correlation between Zn and Fe suggests the possibility of combining both traits. Bio-fortified wheat with superior agronomic performance and durable resistance to all three rusts (including the Ug99 group of races of stem rust fungus) and better resistance to the recently emerged wheat blast are targeted for commercialization in the key wheat Mega-Environments (ME) of 1 and 5 in South Asia, where more than 26% of the population have inadequate Zn intake. Current breeding efforts for enhanced Zn and Fe have focused on transferring genes governing increased Zn from *Triticum spelta*, *T. dicoccon*, *Aegiolops tauschii*-based synthetics, landraces, and other reported high Zn and Fe sources to high-yielding elite wheat backgrounds (Velu et al., 2012). The new variety *Bari Gom 33* was a simple cross between Kachu and Solala. Kachu is a ‘Kauz’-derived high-yielding wheat variety carrying a 2NS segment for blast resistance. The presence of the 2NS translocation in wheat can effectively resist the wheat blast (e.g., Cruz et al., 2016). *Kachu* was released in India in 2010 occupying a considerable share of the North-Western Plains Zone of India. *Solala* is derived from a CIMMYT's pre-breeding cross, involving a durum wheat derivative of *T. polonicum* with long spikes (CMH74A.630/SUPER X//CIANO T 79/3/IRENA). Yield performance of Kachu/Solala and its sister lines ranged from 5.9 to 7.7 t/ha with an average grain yield of 6.6 t/ha under late sowing in Mexico. Few entries had a yield performance similar to or higher than the average of two commercial checks (Roelfs F2007 and Waxwing), suggesting the possibility of combining high-grain yield and enhanced Zn concentration in elite wheat backgrounds.

Based on the grain-yield data, selected entries were analyzed for micronutrient content. Considering the combined data on grain Zn, grain yield, rust resistance, and end-use quality, Kachu/Solala was phenotyped in five artificially managed environments in Ciudad Obregon, Sonora Province of Mexico. In the phenotyping platform, five artificial environments were managed within the same experimental station by varying the planting date and irrigation schedule. For the planting date, seeds were sown early, normal and late with regulated irrigations. In the case of irrigation, usually five irrigations are provided to wheat, but in order to create a water stressed environment, we reduced the number of irrigations to two since most locations in South Asia grow wheat with limited irrigation. The prime objective of generating the five artificially-controlled phenotyping environments was to generate lines suitable for different wheat Mega-Environments (MEs), including ME 5 which is heat stress prone environment with a shorter crop duration (CIMMYT, 2016). Globally, wheat is cultivated in various agroclimatic conditions ranging from hot, humid areas in the lower part of the eastern Gangetic plains to extremely cold areas, such as Canada, Kazakhstan, and Russia. Based on the agroclimatic conditions, biotic and abiotic stresses, rainfall, humidity, temperature, and the major requirements for crop establishment, global wheat-growing areas are divided into 12 MEs (CIMMYT, 2016).

Wheat ME 5 is mainly characterized by low latitude, hot, humid, and mostly irrigated wheat (Fischer et al., 2014). The Obregon experiment station of CIMMYT replicates the ME 5 environment of South Asia. In fact, a large number of CIMMYT wheat varieties have been released in South Asia by direct introduction from Mexico (Mottaleb et al., 2018). On average, about a 5% yield superiority was observed across five environments and in the late-sown heat-stress environment in Obregon, where continuous heat stress occurs during the crop-growing season and higher terminal heat stress towards the end of the crop season. Due to the heat stress, the duration of the crop cycle was reduced by almost a month when compared to optimal planting.

**Fig. 2:**
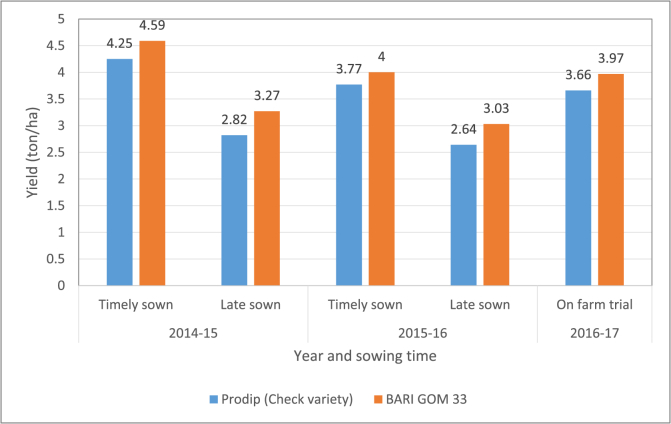
Yield performance of *BARI Gom 33* compared to the check variety *Prodip* over three years 2014–17 in Bangladesh. Notes. Timely sown: 20–30 November. Late sown: 20–25 December. On farm trial was conducted in 7 districts of Bangladesh in the 2016–17 wheat season, with seeds sown November 20 - December 10, 2016.

Source: Prepared based on (BARI, 2017).

Based on the performance in Mexico, Kachu/Solala was included in the 4th HarvestPlus Yield Trial (HPYT) and was distributed to national partners in South Asia, including Bangladesh in 2013–14. This entry showed superior performance across locations for grain yield, a 6–8 ppm Zn increment over local checks, and a large grain size (50 g 1000-grain weight). The superior performance was also observed at Gazipur, Bangladesh and Banaras Hindu University (BHU), Varanasi in the eastern Gangetic plains of India. Interestingly, Kachu/Solala was included in the Participatory Variety Selection (PVS) trials conducted by BHU as BHU-35 (Prof. V.K. Mishra, BHU, Varanasi, *personal communication*). In Bangladesh, during the field trials of 2014–15, 2015–16 and 2016–17 wheat seasons, the new wheat variety consistently provided 5–8% more yield than the check variety *Prodip* under both optimum and late sown conditions (Fig. 2).

After screening against wheat blast in 2016 and 2017 under field and laboratory conditions in Bangladesh (Jessore), Bolivia (Instituto Nacional de Innovacion Agropecuaria y Forestal, INIAF at Quirisillas and Okinawa), and in the USA (United States Department of Agriculture, Agricultural Research Service, (USDA-ARS), Fort Detrick, Maryland), the variety was found resistant to wheat blast. *BARI Gom 33* carries the 2NS segment for blast resistance confirmed by marker analysis. In the multilocation trial in Bangladesh, 408 genotypes from various sources along with *BARI Gom 33* and checks were evaluated against wheat blast under field conditions (Roy et al., 2018b). Out of a disease severity index range from 0 (no blast incidence) to 100 (100% affected by the blast), *BARI Gom 33* showed only 1.45% disease severity and hence was considered resistant to wheat blast (Roy et al., 2018b). The resistance of *BARI Gom 33* was stably expressed under varying dates including optimum to late sown conditions (Roy et al., 2018a). Moreover, the new wheat is moderately resistant to Helminthosporium leaf blight and leaf-rust, which are the most common wheat diseases in Bangladesh (BARI, 2017). Finally, the new wheat is zinc (Zn) enriched (40–45 ppm) in the grains with a Zn advantage of 7–8 ppm over local varieties (BARI, 2017).

Due to better grain yield and resistance to wheat blast, in the 93rd Seed Board meeting of Bangladesh on October 11, 2017, the Bangladesh government approved the new wheat under the name *BARI Gom 33* for release. The complete development and approval process of *BARI Gom 33* is depicted in Table 2.

**Table 2:** Development pathway of wheat variety *BARI Gom 33,* Bangladesh.

Year	Generation	Place, country	Selection/check	Comments
2010	F_1_	El Batan, Mexico	Agronomic performance	
2010–11	F_2_	Obregon, Mx	Leaf rust & good agronomic type	Selected bulk
2011	F_3_	Toluca, Mx	Yellow rust, Septoria & good agronomic type	Individual spikes
2011–12	F_4_	Obregon, Mx	Leaf rust & good agronomic type	Head-rows in Zn area
2012–13	F_5_	Toluca, Mx	Yellow rust, Septoria tritici blotch resistant, plump and large grain	Bulk
2013–14	F_6_	Obregon, Mx	Grain yield, large and plump grain, Zn and Fe concentration	Standard yield trial plots, Zn and Fe measurement with bench top XRF machine
2014	F_7_	El Batan, Mx	Pre-Mexicali seed multiplication	Bulk
2014–15		Gazipur, Bangladesh; Varanasi, India; Obregon, Mx	Yield & yield stability, Zn and Fe content	Superior performance for grain yield (5–8% over the check) and 6–8 ppm Zn increment over local checks. Large grain size (50 g per thousand kernels).
2013–16		El Batan, Mx	End-use grain quality check	Strong and balanced gluten and other essential end-use quality traits to make flatbread in South Asia (chapatti) and yeast bread.
2016–17		Bangladesh, Bolivia & USA	Field and greenhouse screening for blast resistance	Under field condition in Bangladesh and Bolivia, under greenhouse condition in the USA. Almost no infection found. In USDA-ARS, Fort Detrick, USA, WB evaluation performed in the Bio-safety Level-3 Containment greenhouses, with spray inoculation and evaluation based on average percentage of infected spikelets. Had values of 11.1 and 5.4%; whereas the susceptible check, Glenn was completely bleached (severity value 100%), where severity values less than 20% were taken as resistant.
October 11, 2017,		93rd Board meeting, National Seed Board, Bangladesh, approved the variety as *BARI Gom 33* for release in Bangladesh		

Source: Authors and (BARI, 2017).

## 3 Materials and methods

### 3.1 Identifying the wheat blast-vulnerable area

The emergence of wheat blast was first officially recognized in Bangladesh in March 2016, affecting eight districts: Chuadanga, Meherpur, Jessore, Jhenaidah, Bhola, Kushtia, Barisal and Pabna (Islam et al., 2016, Fig. 3). The severity of the wheat blast damage was highest in a sub-set of districts: Bhola, Chuadanga, Jhenaidah and Meherpur (Kabir et al., 2016). In this study, we developed a climate analogue tool by matching the long-term temperature and rainfall patterns (1960–1990) in the four most severely affected districts with other districts of Bangladesh. Note that there is no high-resolution data available for recent years; however, for future work, we plan to use WorldClim 2 datasets which provides very high-resolution climate data (Fick and Hijmans, 2017). The climate analogue approach employed in our study has been utilized for a number of studies focusing on climate change analysis (Berry et al., 2014; Burke et al., 2009; Kellett et al., 2015; Leibing et al., 2013; Pugh et al., 2016; Thornton et al., 2011; Webb et al., 2013).

**Fig. 3:**
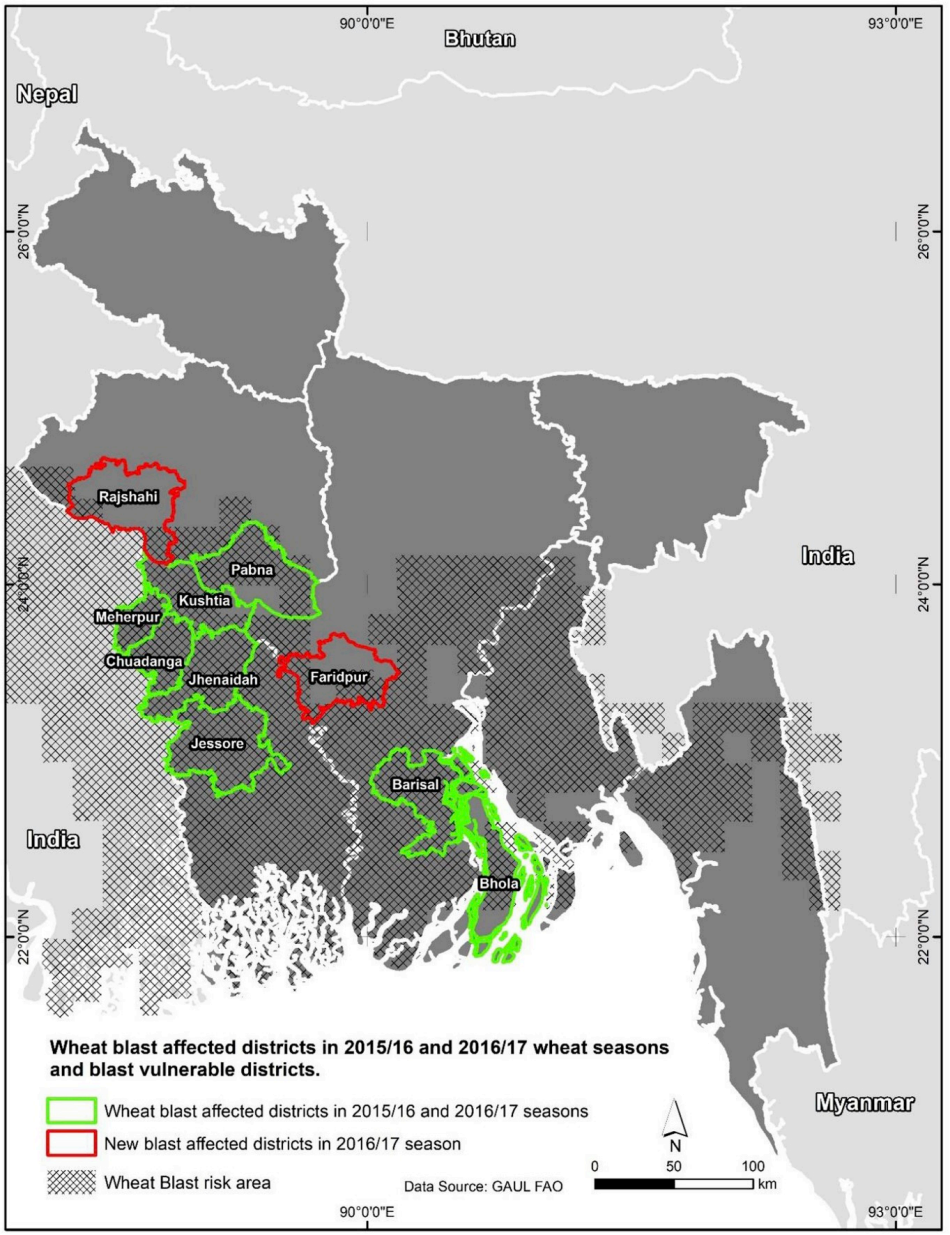
Wheat blast vulnerability in Bangladesh.

Source: Authors' based on Sources: CIMMYT (2017) and Islam et al. (2016).

The analog tool used in the present study is based on an R code originally developed by Ramírez-Villegas et al. (2011), and the code is available in an online archive (Google Code Archive, 2019). It analyzes climate variables at a given location considering rainfall and average temperatures as factors (in combination or for each factor separately) and uses spatial analysis to identify areas in the target zones that have a similar (analogue) climate, based on a weighted similarity index. This analysis can be performed with the current climate data to identify current spatial climate analogues as well as with predicted future climate data, be it looking forward or backward to identify either the future climate of a location or where future predicted climates could be found in Ramírez-Villegas et al. (2011).

Wheat blast is mainly a spike disease, and warm and humid weather during heading generates a conducive environment for the outbreak of the disease. A temperature of 300C with an increased duration of spike wetness has the highest blast incidence in laboratory experiments (Cardoso et al., 2008). Usually, wheat in Bangladesh is planted in November–December and harvested from March to mid-April (BBS, 2018a). The crop comes to heading in January–February. Therefore, weather elements of January–February are the most critical for the emergence of wheat blast in Bangladesh. Considering this fact, we identified spatial analogue areas based on January's and February's weather variables. The gridded outputs for all locations with similarity indices above 0.6 (60%) were merged in a GIS and used to identify wheat-producing districts at risk.

Matching with the weather in the four districts most severely affected by wheat blast (Kabir et al., 2016) at a 60% similarity level, this study identified a total of 232,667 ha wheatland (Triennium Ending [TE] 2017–18) spread over 45 districts in Bangladesh as vulnerable to wheat blast (Table 1; Fig. 3). The vulnerable area represents more than 57% of Bangladesh's total wheat-producing area (403,667 ha, TE, 2017–18) with a similar share (57%) in Bangladesh's wheat production. The wheat blast-vulnerable areas in the 45 districts will be used as one of the key *ex-ante* assessment scenarios and contrasted with the current 10 wheat blast-affected districts, as well as all of Bangladesh's wheat-producing areas.

### 3.2 Potential benefits of *BARI Gom 33*

The new wheat *BARI Gom 33* can provide two types of yield benefits. First, as an improved variety coming out of the breeding pipeline, it presents a 5–8% yield gain over the existing varieties (BARI, 2017, Fig. 2). However, these yield gains were reported under research-managed trials, and we assume a yield adjustment of 80% for farmer fields (CIMMYT, 1988), which for the mid-point would yield an assumed 5.2% yield increase in farmers’ fields. Second, the blast-resistant trait of the variety will avoid yield losses in the case of such biotic stress.

According to Islam et al. (2016), in the 2015–16 wheat season, eight districts of Bangladesh: Pabna, Kushtia, Meherpur, Chuadanga, Jhenaidah, Jashore, Barisal and Bhola, were affected by wheat blast. The most severely affected districts were Meherpur and Chuadanga (40–70% of the wheat-producing area) while the least affected was Pabna (0.2%). As wheat in Bangladesh is produced in almost all districts except a few coastal and hilly districts (Mottaleb et al., 2019a), we constructed district level pooled data consisting of wheat yield (ton/ha), monthly average maximum temperature (^0^C) in January and February, and the monthly total rainfall (millimeters) in January and February from 2010–11 to 2016–17. To predict the yield gain from the blast-resistant traits of *BARI Gom 33* (through mitigation of blast-induced yield loss)*,* we have applied the Ordinary Least Square estimation procedure in a difference-in-difference estimation setting in the pooled dataset. The details of the dataset, the corresponding Stata do file, and the descriptions are publicly available (Annex A). To estimate the highest and lowest bounds of the blast-induced wheat yield loss in the 2015–16 wheat season, we then specify the following function:

(1) $$\begin{array}{l} {Yield_{DT}=\beta_{0}+\beta_{1}(Monthly\;average\;maximum\;temparature\;\left(C\right)\;in\;Janurary)}_{\text{DT}}\\ {+\beta_{2}\;(Monthly\;average\;maximum\;temparature\;\left(C\right)\;in\;February)}_{\text{DT}}\;\\ {+\beta_{3}(Total\;rainfall\;\left(millimeter\right)in\;Janurary)}_{\text{DT}}\\ {+\beta_{3}(Total\;rainfall\;\left(millimeter\right)\;in\;February)}_{\text{DT}}\\ {+\beta_{4}\;\left(2015-16\;wheat\;season\;dummy\right)+\;\beta_{5}\;\left(Meherput\;and\;Chuadanga\;districts\;dummy\right)}_{\text{dH}}\;\\ {+\;\beta_{6}\;\left(Meherput\;and\;Chuadanga\;districts\;dummy\;X\;2015-16\;wheat\;season\;dummy\right)+\;\beta_{7}\;\left(Pabna\;districts\;dummy\right)}_{\text{dL}}+\beta_{8}\;\left(Pabna\;districts\;dummy\;X\;2015-16\;wheat\;season\;dummy\right)\varepsilon_{DT} \end{array}$$

In Equation (1), $\beta_{0}\;$ is a scalar parameter which is an intercept, and $\beta_{i}$ are the parameters to be estimated; and ε is the random error term. Among others, $\beta_{4}$, captures the yield trend in 2015–16 – the year when wheat blast first emerged in Bangladesh, compared to all other sampled years; $\beta_{5}$, captures the general yield trend in Meherpur and Chuadanga districts, and $\beta_{6}$ is our difference-in-difference (DiD) estimator, that captures any change in the yield in 2015–16 in the severely blast-affected Meherpur and Chuadanga districts. In this study, we treat $\beta_{6}$ as the upper limit of the wheat blast induced yield loss in Bangladesh. Analogically, $\;\beta_{7}$ captures the general yield trend in Pabna district, and $\beta_{8}$ is our DiD estimator for Pabna, the least blast-affected district in 2015–16. $\beta_{8}$ is treated as the estimated lower limit of the wheat blast induced yield loss in Bangladesh.

In Table 3, we have presented two different estimated models separately for the least affected district Pabna and the most severely affected districts Chuadanga and Meherpur. Columns a1 and b1 present the intercept and dummy variables only models, and columns a2 and b2 present the full model that includes all other possible explanatory variables. The estimated functions in Table 3 shows that on average, in 2015–16, wheat yield was higher by 90 kg-140 kg/ha compared to the all sampled years $\left(\beta_{4}\right)$, and in particular wheat yield in Meherpur and Chuadanga districts was higher by 910 kg-960kg than any other districts, on average ($\beta_{5}).$ Similarly, on average wheat yield in Pabna district was higher by 630 kg-750 kg/ha. $\left(\beta_{7}\right).$

**Table 3:** Estimating yield loss applying Ordinary Least Square estimation procedure separately in the least blast-affected and the most severely affected districts of Bangladesh in the 2015–16 wheat season.

	Estimated yield loss in the least blast-affected district, Pabna		Estimated yield loss in the most severely blast-affected districts, Meherpur and Chuadanga	
Model	a1	a2	b1	b2
Dependent variable	Yield (ton/ha)			
Independent variables				
Monthly average maximum temperature (^0^C) in January ($\beta_{1}$)		−0.12* (0.06)		−0.11* (0.06)
Monthly average maximum temperature (^0^C) in February ($\beta_{2}$)		−0.05 (0.06)		−0.06 (0.06)
Monthly total rainfall (mm) in January ($\beta_{3}$)		0.01*** (0.00)		0.01*** (0.00)
Monthly total rainfall (mm) in February ($\beta_{4}$)		0.002 (0.00)		0.001 (0.00)
Year 2015–16 dummy (yes = 1) when blast emerged ($\beta_{5}$)	0.09*** (0.03)	0.11 (0.08)	0.10*** (0.03)	0.14* (0.08)
Meherpur and Chuadanga district dummy (yes = 1) ($\beta_{6}$)			0.96*** (0.11)	0.91*** (0.10)
Meherpur and Chuadanga district dummy X Year 2015–16 dummy ($\beta_{7}$)			−0.55*** (0.05)	−0.51*** (0.12)
Pabna district dummy (yes = 1) ($\beta_{8}$)	0.75*** (0.10)	0.63*** (0.07)		
Pabna district dummy X Year 2015–16 dummy ($\beta_{9}$)	−0.03 (0.03)	−0.08 (0.11)		
Observations	447	447	447	447

Note: Values in parentheses are robust standard errors. *Significant at the 10% level, ** Significant at the 5% level and ***Significant at the 1% level.

A1 and b1: Intercept and dummy only models.

A2 and b2: Full model with all possible explanatory variables.

Blast-induced yield loss in 2015–16 = {80 (model a2) + 510 (model b2)}/2 = 295 kg/ha.

Yield gain from the blast-resistant trait (assuming 90% mitigation) = 295 × 0.90 = 266 kg/ha.

Source: Authors’ estimation based on data described in Annex A.

Our DiD estimators ($\beta_{6}and\;\beta_{8}$), demonstrate that in the year 2015–16, the blast induced wheat yield loss was 510 kg-550 kg/ha in Chuadanga and Meherpur districts ($\beta_{6}$), and in Pabna district it was lower by a non-significant 30 kg-80 kg/ha in 2015–16. As *BARI Gom 33* was found resistant in >95% cases (Roy et al., 2018b), we assumed that *BARI Gom 33* could mitigate the blast induced yield loss by {(510 + 80)/2} × 0.90 = 266 kg/ha conditional on a potential outbreak of the disease in the currently 10 blast-affected districts, or in the 45 wheat blast-vulnerable districts (Table 3). Among other variables, the monthly average maximum temperature in the month of January ($\beta_{1}$) generates a significantly negative impact on wheat yield in Bangladesh, whereas rainfall in January ($\beta_{3}$) positively and significantly affects wheat yield (Table 3).

The bread quality and taste of *BARI Gom 33* is the same as any other popular wheat in Bangladesh. The zinc-enriched trait is a largely-invisible trait and is not expected to generate a price premium in the market (see e.g. Hellin and Erenstein, 2009). The trait could still have significant positive additional impacts on the nutritional status of Bangladeshi consumers. This is because, although Bangladesh is highly successful in maintaining food security by achieving self-sufficiency in rice production, 41% of children under five years old are chronically undernourished, and therefore stunted (WFP, 2015). Also, one-third of children between six months and five years old are anemic, while 24% of the women in Bangladesh are underweight (WFP, 2015). Unfortunately, the overall costs of undernourishment in Bangladesh due to productivity loss is USD 1 billion per year (WFP, 2015). The benefits of bio-fortification can potentially be estimated through, for instance, the Disability-Adjusted Life Years (DALY) framework (Meenakshi et al., 2010). However, for simplicity's sake, we only account for the yield benefits and assume bio-fortification would potentially generate additional benefits on top of what is being presented.

We retain two simulation scenarios:

− Scenario 1: The new variety expresses (only) a yield gain (5.2%) in the absence of blast incidence, i.e. there is no additional averted yield loss due to wheat blast. As the potential blast induced yield loss in susceptible varieties, and equivalently the gain from the blast-resistance traits of the new wheat is conditional on the outbreak of the wheat blast disease, there may be no such yield loss averted if there is no such outbreak.
− Scenario 2: The new variety expresses both a yield gain (5.2%) and an averted yield loss (0.266 ton/ha) due to wheat blast. This assumes wheat blast losses at a similar rate as 2015–16 with yield effects as elaborated above.

The corresponding yield gains of *BARI Gom 33* under scenarios 1 and 2 for different geographical-based scenarios are presented in Table 4.

**Table 4:** Calculation of yield scenarios considering 5.2% yield gain and 266 kg/ha yield gain from the blast-resistant traits of *BARI Gom 33*.

Base yield and estimated gains				Simulated yield scenarios	
	Base yield (ton/ha)^a^	5.2% yield gain from *BARI Gom 33*	0.266 ton/ha yield gain from the blast-resistant trait	Scenario 1	Scenario 2
				Actual yield + 5.2% yield gain	Actual yield + 5.2% yield gain+ 0.266 ton/ha gain from the blast-resistant trait
	a	a+b	c	a+b	a + b+ c
10 currently blast-affected districts	3.12	0.162	0.266	3.28	3.55
45 blast-vulnerable districts	2.65	0.138	0.266	2.79	3.05
National	3.11	0.162		3.27	

a Triennium average ending 2016–18. Sources: Authors' calculations based on BBS (2014; 2016; 2017a).

### 3.3 Adoption scenarios

The adoption of new agricultural technology, including improved seeds, is seldom instantaneous (Pierpaoli et al., 2013). For one, the new technology needs to be available to farmers to be potentially adopted. A new variety needs to have its seed bulked up, often initiated only after its formal release. Furthermore, technology adoption is a complex process influenced by a large number of factors (Gershon et al., 1985; Mottaleb, 2018; Rogers, 1983), risks and uncertainties about the proper application, suitability with the environment and farmers’ expectations (World Bank, 2008). As a result, the adoption process can be slow (World Bank, 2008), and the entire adoption process may fail in the absence of strong public support and extension services.

We assumed that in 2017–18, the initial 400 kg of breeders' seed of *BARI Gom 33* would be cultivated on 2.9 ha of land (seed rate @140 kg/ha) and the cultivation cost would be USD800/ha. For simplicity, we assumed a standard seed rate at 140 kg/ha as it is the recommended seed rate for *BARI Gom 33* for the highest yield performance (BARI, 2017), although in practice seed rates may vary somewhat (e.g. by agroecology and farmers’ preference). Wheat multiplication follows 1:20 to 1:25 coverage, which means one ha of wheat production under new seed can generate seeds for 20–25 ha for the next season (Joshi et al., 2011; Tamil Nadu Agricultural University, 2014). In this study, we have assumed 1:20 coverage so that a minimum expected prediction is realized. Under this assumption in the 2018-19 season, a total of eight tons of seeds will be available and cultivated on 57.1 ha of land. Finally, in the 2019-20 season, 160 tons of seed of *BARI Gom 33* will be available to farmers for cultivation on 1142.9 ha of land, which is 0.8% of the total wheat area of the 10 wheat blast-affected districts, 0.4% of the total 45 wheat blast-vulnerable districts, and nearly 0.3% of the total wheat-growing area of Bangladesh.

Assuming a logistic adoption and diffusion process, and considering the seed multiplication rate, it is assumed that by 2024 the new wheat will be cultivated on 24% of the wheat area of the sampled areas and throughout Bangladesh. By 2027, the adoption rate will reach a maximum of 30% and plateau thereafter until 2029–30. The adoption process we have assumed is plausible, as empirical evidence shows that, if new crop varieties are effectively resistant to the emerging biotic and abiotic threats, farmers will respond by rapidly adopting such varieties. For example, with the collaboration from CIMMYT, INIFAP, Mexico developed CIRNO 2008 wheat variety that is resistant to leaf-rust disease and provided a 12% yield gain over the existing durum wheat varieties in Mexico (Figueroa-López et al., 2010). Leaf-rust disease was a severe yield-deterring factor in Yaqui Valley, Sonora State, Mexico. The CIRNO 2008 variety was released in 2008, and it was cultivated on 29 ha of land in the initial year. By 2016, the variety occupied 147,342 ha out of 235,000 ha of land in Yaqui valley, i.e. an adoption rate of more than 65%. Based on the empirical evidence, the assumption of a 30% adoption rate in eight years in Bangladesh is a more conservative assumption.

### 3.4 Other assumptions

In addition to the adoption assumptions, the following major assumptions are considered in the benefit quantification process:

- the new wheat will continue to be available for seed multiplication and in the hands of the farmers by the 2019–20 wheat season; - the seed production, multiplication, and dissemination costs amount to USD 800/ha. The per hectare seed production cost is based on Lagos and Hossain (2016), which mentioned that the wheat production cost in Bangladesh is USD 663/ha. As seed production cost is higher in general than wheat grain production cost considering that the seed quality must be higher than the ordinary grain produced for consumption, we add an extra 21% of the current wheat production cost per ha (USD 137), and assumed the seed production and dissemination cost will be USD 800/ha
- the government will support seed production and dissemination cost (@USD 800/ha) during the initial four years and thereby invest USD 3.9 million (2017–20 @ 5% discount rate) if only in 10 blast-affected districts; USD 4.3 million in 45 blast-vulnerable districts; and USD 11.7 million for the whole of Bangladesh. The aggregate dissemination cost was estimated as adoption rate × total wheat area (ha) × USD 800 in a single year. In Bangladesh, the farmer-to-farmer seed diffusion process is highly effective in disseminating new variety. For example, Pandit et al. (2011) demonstrate that *BARI Gom 26,* Ug99 tolerant wheat, was released in Bangladesh in 2010 with 252 kg initial seeds. After three years, the total seed production of this new wheat was 19.4-ton, which reached to 969 farmers in 24 villages completely through the farmer-to-farmer informal seed dissemination process (Pandit et al., 2011). We thus assume that the farmer-to-farmer seed diffusion process will continue to propel the dissemination of the new wheat after the initial investment of the government
- the domestic market price of wheat is set at a constant USD 253/ton for the whole simulation period, based on the average harvest time wheat price in Bangladesh (triennium ending 2018, Table 1)
- no intrinsic wheat yield growth rate or other technical change are considered in this study.

Considering the future stream of net benefits and costs, the net present value (NPV) from the dissemination of new wheat was calculated as follows:

$$\text{NPV}\;=\;\left\{\frac{\sum_{t=2017}^{2030}Revenue\;from\;new\;wheat\;variety\;-seed\;mulitiplication\;and\;dissimination\;costs}{{\left(1+r\right)}^{t}}\right\}$$

where r is the discounting factor, which is assumed to be 5% based on the fact the bank rate in Bangladesh in 2017 and 2019 was fixed at 5% (Bangladesh Bank, 2019), and *t,* is the number of sampled years which is 14 (2017–2030) in our case.

## 4 Major findings: economic quantification

The economic benefits of the dissemination of *BARI Gom 33* in Bangladesh are presented in Table 5. The detail of the calculation process and the corresponding excel sheets are publicly available (Annex B). In the 10 districts that were affected by wheat blast, 120,666 ha (triennium average ending 2018) of wheat-producing land was affected, which was nearly 30% of the total wheat-producing land of the country (Table 1). The actual wheat production in these 10 districts was 379.3 thousand metric tons (triennium average ending 2017–18), which was more than 30% of the total 1.3 million tons of wheat that was produced (triennium ending 2018) in Bangladesh (Table 1). As the government is discouraging wheat cultivation in the blast-affected districts since the emergence of wheat blast (Mottaleb et al., 2019a), the wheat production in these districts and consequently in all of Bangladesh has been declining since 2015–16 (Table 1).

**Table 5:** Net present value (NPV) of the future stream of benefits of dissemination of BARI Gom 33 in Bangladesh, 2016–17 to 2029–2030.

Target area	Scenario 1: Current actual yield + 5.2% yield gain				Scenario 2: Current actual yield + 5.2% yield gain +0.266 ton/ha gain from blast resistance traits			
	Production gain (000, ton)	Discounted market value of the gain (million USD)	Discounted seed cost (million USD)	NPV (million USD	Production gain (000, ton)	Discounted market value of the gain (million USD)	Seed cost (million USD)	NPV (million USD
In 10 currently blast-affected districts	46.5	7.2	3.9	3.20	123.0	18.9	3.9	14.9
45 wheat blast-vulnerable districts	68.8	10.4	4.3	4.81	196.4	29.6	4.3	21.7
All wheat areas in Bangladesh	135.6	20.4	11.7	8.7				

Source: Authors' calculations.

Under the assumed adoption rate of *BARI Gom 33* at a maximum of 30% in 2027, and assuming a yield gain of 5.2% from the new wheat (expected yield 3.28 ton/ha), the total cumulative production from the *BARI Gom 33* covered area will be 46.5 thousand metric tons (Tables 6a–8, Annex B). If the entire wheat-producing area of the 10 wheat blast-affected districts were under entirely traditional varieties (no adoption of *BARI Gom 33*), the total production would be 5.28 million tons (average yield in the blast-affected districts (3.12 ton/ha) X total area in the blast-affected districts). This shows that the net cumulative production gain of the dissemination of the new wheat would be 46.5 thousand metric tons, worth USD 7.2 million (after being discounted at a 5% rate) at the harvest time market price of wheat, USD 253/metric ton.

Considering the seed multiplication and dissemination costs in the first four years of USD 3.9 million, our calculation shows that, at a 5% discount rate, the dissemination of the new wheat only in 10 wheat blast-affected districts can generate USD 3.23 million of net gain with a yearly average return of USD 0.23 million (Scenario 1, Table 5). Under the assumption of a potential outbreak of wheat blast and, therefore, with the gain from the blast-resistant traits of the new wheat (expected yield will be 3.55 ton/ha), the net production gain would be 123 thousand tons from the adoption of the new variety that provides a 5.2% yield gain and a 0.266 ton/ha yield gain due to blast-resistant traits, worth USD 18.9 million (at a 5% discount rate). Following the assumption of the dissemination costs of USD 3.9 million in the initial four years, the NPV could be USD 14.9 million (Scenario 2, Table 5), with a yearly average return of USD 1.1 million.

Considering the dissemination of the new wheat that provides a 5.2% yield gain in 45 wheat blast-vulnerable districts, our simulation exercise shows that the cumulative net production gains from the new wheat by 2030–31 would be 68.8 thousand metric tons, worth USD 10.4 million (Scenario 1, Table 5). Considering the total seed multiplication and dissemination costs in the first four years of USD 4.3 million, the net benefit at a 5% discount rate of the dissemination of the new wheat in 45 wheat blast-vulnerable districts would be USD 4.8 million with a yearly average return of USD 0.34 million (Scenario 1, Table 5). In case of a potential wheat blast outbreak (Scenario 2), the net production gain would be 196 thousand tons from the adoption of the new variety that provides a 5.2% yield gain plus 0.266 ton/ha due to the blast-resistant trait worth USD 29.6 million (Table 5). The NPV of the net benefit would be USD 21.7 million with a yearly average return of USD 1.6 million (Table 5).

Considering the dissemination of the new wheat that provides 5.2% yield gain for all of Bangladesh, the cumulative net production gains from the new wheat between 2017 and 2030 would be 135.6 thousand metric tons, worth USD 20.4 million. Under the assumption of the seed development and dissemination costs of USD 800/ha, the total seed multiplication and dissemination cost would be USD 11.7 million, and the net present value of the benefit at a 5% interest rate would be USD 8.7 million with a yearly average return of USD 0.62 million (Scenario 1, Table 5). Note that the gain from the blast-resistant trait of the new wheat may offer extra yield gain at the time of the outbreak of blast, and it is feasible to assume that wheat blast cannot affect the entire wheat-growing area of Bangladesh. Hence, in this simulation exercise, we have not considered the gain from the blast-resistant traits of the new wheat considering the dissemination assumption throughout Bangladesh.

It is important to mention here that the *ex-ante* impact assessment process applied in this study is a consumer surplus analysis by nature in a partial equilibrium setting. Any change in any crucial parameter, such as the price of wheat, the dissemination cost, and the discount rate, can change the ex-ante return level of NPV significantly.

## 5 Conclusions and policy implications

Wheat blast is a devastating seed-borne and wind-borne pathogen; hence, the rapid multiplication and dissemination of blast-resistant wheat seed is crucial to combat the disease. To control wheat blast, BARI with the technical support of CIMMYT has developed a new blast-resistant, high yielding, zinc-fortified wheat, and the national seed board of Bangladesh approved the new wheat for dissemination in 2017. Under the existing dissemination process, it will take four to five years for the new wheat to reach the hands of farmers. This indicates the need for external support to speed up the dissemination process of the new seed. As such seed multiplication and dissemination entails costs, the present study examined the potential net economic benefits of the dissemination of the new wheat first in the currently blast-affected 10 districts, second in the 45 wheat blast-vulnerable districts, and finally throughout Bangladesh.

This study presented the ex-ante economic benefits of the dissemination of the newly approved *BARI Gom 33*, a blast-resistant high-yielding wheat in Bangladesh. Findings of this study demonstrated that whether or not wheat blast is a threat to the current wheat production in Bangladesh, the dissemination of the new zinc-fortified high-yielding wheat could bring substantial economic gains, as the new wheat provides a 5.2% more yield gain than the existing varieties. However, due to the blast-resistant trait of the new wheat, the dissemination of *BARI Gom 33* can bring higher net benefits in the case of a potential outbreak of wheat blast. As wheat blast initially emerged in eight districts in 2015–16 and, despite the unfavorable climatic conditions for its development, the disease was seen in an additional two districts in 2016–17, it is now an established disease in Bangladesh. Alarmingly, our climate analogue model analysis identified a total of 232,666 ha of wheat areas (Triennium Ending [TE] 2017–18) in 45 districts (Table 1) as vulnerable to wheat blast, which is more than 57% of Bangladesh's total wheat-producing areas of 403,666 ha (TE, 2016–18). Therefore, the dissemination of the new blast-resistant wheat can be a major instrument to mitigate the wheat blast threat in Bangladesh.

Our simulation exercise shows that the benefits from the new wheat far exceed the seed multiplication and dissemination costs even if we consider only a 5.2% yield gain of the new wheat variety. The benefits from the new wheat would be nearly double in the case of a potential outbreak of wheat blast in currently blast-affected areas, as well as blast-vulnerable districts. Based on the findings, the present study urges international development organizations and donor agencies to support the national government's efforts to disseminate the new blast-resistant, zinc-enriched and high-yielding wheat in Bangladesh to effectively combat the wheat blast threats. Note that the investment in Bangladesh to combat wheat blast through the rapid dissemination of seed can have positive externalities to neighboring countries, such as India, which is a major producer, consumer, and an emerging exporter of wheat, and alarmingly 21% of its 30.96 million ha of wheat is vulnerable to wheat blast (Mottaleb et al., 2018b). Also, as the new wheat variety is zinc fortified, the rapid dissemination of the new wheat can also play a significant role in mitigating malnutrition.

This study points out that to begin the adoption and diffusion process of *BARI Gom 33* in the 2019–20 wheat season, the minimum seed requirement will be 160 metric tons, which will require a nearly USD 1 million initial investment in seed multiplication from the 2017–18 to the 2019-20 seasons. To speed up the seed multiplication process, the Bangladesh government may consider seed imports from India, Mexico, or other suitable countries under a complete and strict quarantine process and treatment. International donor agencies and research institutes such as CIMMYT can play an important role in this case. Finally, the dissemination of new wheat technologies, including high-yielding and blast-resistant traits would allow the resource-poor farmers to better cope with the new threats and changing agro-climatic conditions. Thus, this study strongly suggests that donor agencies fund the product development pipeline and dissemination of new seed varieties that are tolerant to new and emerging deadly biotic and abiotic threats.

## Conflicts of interest

The authors declare no conflict of interest.

## Figures and Tables

**Table 6a:** Ex-ante benefits of dissemination of new wheat in 10 blast-affected districts in Bangladesh, Scenario 1: current yield +5.2% yield gain

Sampled years	Adoption rate	Area	Actual yield	Actual yield +5.2% yield gain	Simulated production from the area under new wheat (ton)	Production from the area under old wheat (ton)	Total production if the entire area was under old wheat (ton)	Changed production (ton)	Wheat price (USD/ton)	Market value of the increased wheat produced (USD)	Seed multiplication and dissemination costs (USD 800/ha)	Net benefit
2016–17	0	120966.7	3.12	3.28	0	377416	377416	0	253	0	1000000	−1000000
2017–18	0	120966.7	3.12	3.28	0	377416	377416	0	253	0	1000000	−1000000
2018–19	0	120966.7	3.12	3.28	0	377416	377416	0	253	0	1000000	−1000000
2019–20	0.015	120966.7	3.12	3.28	6011	371702	377416	297	253	75063	1465148	−1390085
2020–21	0.058	120966.7	3.12	3.28	22954	355595	377416	1133	253	286652		286652
2021–22	0.115	120966.7	3.12	3.28	45599	334068	377416	2251	253	569443		569443
2022–23	0.185	120966.7	3.12	3.28	73505	307539	377416	3628	253	917939		917939
2023–24	0.242	120966.7	3.12	3.28	96150	286012	377416	4746	253	1200730		1200730
2024–25	0.275	120966.7	3.12	3.28	109082	273718	377416	5384	253	1362231		1362231
2025–26	0.285	120966.7	3.12	3.28	113052	269944	377416	5580	253	1411811		1411811
2026–27	0.3	120966.7	3.12	3.28	119104	264191	377416	5879	253	1487382		1487382
2027–28	0.3	120966.7	3.12	3.28	119104	264191	377416	5879	253	1487382		1487382
2028–29	0.3	120966.7	3.12	3.28	119104	264191	377416	5879	253	1487382		1487382
2029–30	0.3	120966.7	3.12	3.28	119104	264191	377416	5879	253	1487382		1487382
							5283824	46535		7159071	$3,928,629	

At 5% discount rate: Dissemination cost: USD 3.9 million; Net present value (NPV), USD 3.23 million; yearly average return USD 0.23 million.

**Table 6b:** Ex-ante benefits of dissemination of new wheat in 10 blast-affected districts in Bangladesh, Scenario 2: current yield +5.2% yield gain +0.266 ton/ha yield gain from the blast resistant trait

Sampled years	Adoption rate	Area	Actual yield	Actual yield +5.2% yield gain	Actual yield +5.2% yield gain+ 5.1% yield gain from blast resistant trait	Simulated production from the area under new wheat (ton)	Production from the area under old wheat (ton)	Total production if the entire area was under old wheat (ton)	Changed production (ton)	Wheat price (USD/ton)	Market value of the increased wheat produced (USD)	Multiplication and dissemination costs (USD 800/ha)	Net benefit
2016–17	0	120966.7	3.12	3.28	3.55	0	377416	377416	0	253	0	1000000	−1000000
2017–18	0	120966.7	3.12	3.28	3.55	0	377416	377416	0	253	0	1000000	−1000000
2018–19	0	120966.7	3.12	3.28	3.55	0	377416	377416	0	253	0	1000000	−1000000
2019–20	0.015	120966.7	3.12	3.28	3.55	6498	371702	377416	784	253	198315	1465148	−1266833
2020–21	0.058	120966.7	3.12	3.28	3.55	24814	355595	377416	2993	253	757327		757327
2021–22	0.115	120966.7	3.12	3.28	3.55	49294	334068	377416	5946	253	1504454		1504454
2022–23	0.185	120966.7	3.12	3.28	3.55	79462	307539	377416	9586	253	2425172		2425172
2023–24	0.242	120966.7	3.12	3.28	3.55	103943	286012	377416	12539	253	3172299		3172299
2024–25	0.275	120966.7	3.12	3.28	3.55	117923	273718	377416	14225	253	3598982		3598982
2025–26	0.285	120966.7	3.12	3.28	3.55	122215	269944	377416	14743	253	3729969		3729969
2026–27	0.3	120966.7	3.12	3.28	3.55	128757	264191	377416	15532	253	3929626		3929626
2027–28	0.3	120966.7	3.12	3.28	3.55	128757	264191	377416	15532	253	3929626		3929626
2028–29	0.3	120966.7	3.12	3.28	3.55	128757	264191	377416	15532	253	3929626		3929626
2029–30	0.3	120966.7	3.12	3.28	3.55	128757	264191	377416	15532	253	3929626		3929626
								5283824	122945		18914089	$3,928,629	

At 5% discount rate: Dissemination cost: USD 3.9 million; Net present value (NPV), USD 14.9 million; yearly average return USD 1.07 million.

**Table 7a:** Ex-ante benefits of dissemination of new wheat in 45 blast-affected districts in Bangladesh, Scenario 1: current yield +5.2% yield gain

Sampled years	Adoption rate	Area	Actual yield	Actual yield +5.2% yield gain	Simulated production from the area under new wheat (ton)	Production from the area under old wheat (ton)	Total production if the entire area was under old wheat (ton)	Changed production (ton)	Wheat price (USD/ton)	Market value of the increased wheat produced (USD)	Seed multiplication and dissemination costs (USD 800/ha)	Net benefit
2016–17	0	232667	2.65	2.79	0	616567	616567	0	253	0	1000000	−1000000
2017–18	0	232667	2.65	2.79	0	616567	616567	0	253	0	1000000	−1000000
2018–19	0.0004	232667	2.65	2.79	262	616318	616567	13	253	3327	1000000	−996673
2019–20	0.01	232667	2.65	2.79	6650	610250	616567	334	253	84425	1906826	−1822401
2020–21	0.03	232667	2.65	2.79	16386	601003	616567	822	253	208024		208024
2021–22	0.06	232667	2.65	2.79	37531	580919	616567	1883	253	476471		476471
2022–23	0.11	232667	2.65	2.79	74557	545751	616567	3741	253	946524		946524
2023–24	0.19	232667	2.65	2.79	120185	502412	616567	6031	253	1525792		1525792
2024–25	0.24	232667	2.65	2.79	157211	467245	616567	7889	253	1995845		1995845
2025–26	0.27	232667	2.65	2.79	178356	447160	616567	8950	253	2264292		2264292
2026–27	0.30	232667	2.65	2.79	194742	431597	616567	9772	253	2472316		2472316
2027–28	0.30	232667	2.65	2.79	194742	431597	616567	9772	253	2472316		2472316
2028–29	0.30	232667	2.65	2.79	194742	431597	616567	9772	253	2472316		2472316
2029–30	0.30	232667	2.65	2.79	194742	431597	616567	9772	253	2472316		2472316
							8631933	68751		10354883	4291998	

At 5% discount rate: Dissemination cost: USD 4.3 million; Net present value (NPV), USD 4.81 million; yearly average return USD 0.34 million.

**Table 7b:** Ex-ante benefits of dissemination of new wheat in 45 blast-affected districts in Bangladesh, Scenario 2: current yield +5.2% yield gain +0.266 ton/ha yield gain from the blast resistant trait

Sampled years	Adoption rate	Area	Actual yield	Actual yield +5.2% yield gain	Actual yield +5.2% yield gain+ 5.1% yield gain from blast resistant trait	Simulated production from the area under new wheat (ton)	Production from the area under old wheat (ton)	Total production if the entire area was under old wheat (ton)	Changed production (ton)	Wheat price (USD/ton)	Market value of the increased wheat produced (USD)	Multiplication and dissemination costs (USD 800/ha)	Net benefit
2016–17	0	232667	2.65	2.79	3.05	0	616567	616567	0	253	0	1000000	−1000000
2017–18	0	232667	2.65	2.79	3.05	0	616567	616567	0	253	0	1000000	−1000000
2018–19	0.0004	232667	2.65	2.79	3.05	286	616318	616567	38	253	9504	1000000	−990496
2019–20	0.01	232667	2.65	2.79	3.05	7270	610250	616567	953	253	241213	1906826	−1665612
2020–21	0.03	232667	2.65	2.79	3.05	17913	601003	616567	2349	253	594356		594356
2021–22	0.06	232667	2.65	2.79	3.05	41029	580919	616567	5381	253	1361345		1361345
2022–23	0.11	232667	2.65	2.79	3.05	81505	545751	616567	10689	253	2704355		2704355
2023–24	0.19	232667	2.65	2.79	3.05	131385	502412	616567	17231	253	4359405		4359405
2024–25	0.24	232667	2.65	2.79	3.05	171861	467245	616567	22539	253	5702415		5702415
2025–26	0.27	232667	2.65	2.79	3.05	194977	447160	616567	25571	253	6469404		6469404
2026–27	0.30	232667	2.65	2.79	3.05	212890	431597	616567	27920	253	7063760		7063760
2027–28	0.30	232667	2.65	2.79	3.05	212890	431597	616567	27920	253	7063760		7063760
2028–29	0.30	232667	2.65	2.79	3.05	212890	431597	616567	27920	253	7063760		7063760
2029–30	0.30	232667	2.65	2.79	3.05	212890	431597	616567	27920	253	7063760		7063760
								8631933	196431		29585381	4291998	

At 5% discount rate: Dissemination cost: USD 4.3 million; Net present value (NPV), USD 21.7 million; yearly average return USD 1.55 million.

**Table 8:** Ex-ante benefits of dissemination of new wheat in entire Bangladesh, Scenario 1: current yield +5.2% yield gain

Sampled years	Adoption rate	Area	Actual yield	Actual yield +5.2% yield gain	Simulated production from the area under new wheat (ton)	Production from the area under old wheat (ton)	Total production if the entire area was under old wheat (ton)	Changed production (ton)	Wheat price (USD/ton)	Market value of the increased wheat produced (USD)	Seed multiplication and dissemination costs (USD 800/ha)	Net benefit
2016–17	0	403686	3.11	3.27	0	1255462	1255462	0	253	0	1500000	−1500000
2017–18	0	403686	3.11	3.27	0	1255462	1255462	0	253	0	1500000	−1500000
2018–19	0	403686	3.11	3.27	0	1255462	1255462	0	253	0	1500000	−1500000
2019–20	0	403686	3.11	3.27	0	1255462	1255462	0	253	0	1500000	−1500000
2020–21	0.025	403686	3.11	3.27	33321	1223771	1255462	1630	253	412492	8152017	−7739525
2021–22	0.058	403686	3.11	3.27	76321	1182876	1255462	3734	253	944794		944794
2022–23	0.115	403686	3.11	3.27	151614	1111267	1255462	7418	253	1876864		1876864
2023–24	0.185	403686	3.11	3.27	244401	1023020	1255462	11958	253	3025495		3025495
2024–25	0.242	403686	3.11	3.27	319695	951410	1255462	15643	253	3957564		3957564
2025–26	0.275	403686	3.11	3.27	362694	910515	1255462	17747	253	4489867		4489867
2026–27	0.3	403686	3.11	3.27	396016	878824	1255462	19377	253	4902359		4902359
2027–28	0.3	403686	3.11	3.27	396016	878824	1255462	19377	253	4902359		4902359
2028–29	0.3	403686	3.11	3.27	396016	878824	1255462	19377	253	4902359		4902359
2029–30	0.3	403686	3.11	3.27	396016	878824	1255462	19377	253	4902359		4902359
							17576474	135638		20389289	11706244	

At 5% discount rate: Dissemination cost: USD 11.7 million; Net present value (NPV), USD 8.7 million; yearly average return USD 0.62 million.

## Annexure A

Data on yield, temperature, rainfall that we used to estimate the wheat blast-induced yield loss are publicly available online URL: https://data.cimmyt.org/dataset.xhtml?persistentId=hdl:11529/10548178.

Specifically, files: Data description; for reviewer.rar; and Model1.do.

Data on economic quantification and simulation sheets (MS Excel) are publicly available from the URL: https://data.cimmyt.org/dataset.xhtml?persistentId=hdl:11529/10548178.

Specific file: Simulation_revised.rar.
